# Quantitative computed tomography texture analysis: can it improve diagnostic accuracy to differentiate malignant lymph nodes?

**DOI:** 10.1186/s40644-019-0214-8

**Published:** 2019-05-22

**Authors:** So Youn Shin, Il Ki Hong, Yong Suk Jo

**Affiliations:** 1 Department of Radiology, Kyung Hee University Hospital, College of Medicine, Kyung Hee University, Seoul, Republic of Korea; 2 Department of Nuclear Medicine, Kyung Hee University Hospital, College of Medicine, Kyung Hee University, Seoul, Republic of Korea; 3 Division of Pulmonary and Critical Care Medicine, Department of Internal Medicine, KyungHee University Hospital, Seoul, Republic of Korea; 4 Department of Internal Medicine, Division of Pulmonary, Allergy, and Critical Care Medicine, Hallym University Kangdong Sacred Heart Hospital, Seoul, Republic of Korea

**Keywords:** Texture and shape analysis, Mediastinal lymph node, Malignancy

## Abstract

**Background and objective:**

Mediastinal lymph node (LN) staging in individuals with non-small-cell lung cancer plays an important role in staging and treatment planning. This study aimed to assess the accuracy of computed tomography (CT) texture analysis (CTTA) in differentiating benign and malignant mediastinal LNs.

**Methods:**

Pathologically confirmed malignant and benign mediastinal LN samples, obtained using endobronchial ultrasound-guided transbronchial needle aspiration (EBUS-TBNA), were retrospectively reviewed, in addition to chest CT and 18-fluorodeoxyglucose (FDG) uptake positron emission tomography (PET) data. For each LN, CTTA was performed using “AVIEW” software (Coreline Soft, Republic of Korea) by drawing a region of interest.

**Results:**

A total of 132 LNs from 80 patients were included and classified into two groups according to pathology results: malignant (*n* = 61) and benign (*n* = 71). In EBUS, size > 1 cm, round shape, heterogeneous echogenicity, and presence of coagulation necrosis sign were more prevalent in malignant than in benign LNs; length was the only feature that distinguished the two groups. Among CTTA features, compactness and normalized standard deviation (SD) showed differences between the two groups. The ability to distinguish malignant LNs was higher using high standard uptake value (SUV) on FDG PET/CT (SUV_max_ ≥ 5) and normalized SD on CTTA (area under the receiver operating characteristic curve 0.739 versus 0.742, respectively); however, normalized SD demonstrated very low sensitivity despite high specificity.

**Conclusions:**

CTTA may be helpful in distinguishing between benign and malignant LNs; however, the diagnostic value was not high. Therefore, integrated evaluation with other imaging modalities is needed.

**Electronic supplementary material:**

The online version of this article (10.1186/s40644-019-0214-8) contains supplementary material, which is available to authorized users.

## Background

Endobronchial ultrasound-guided (EBUS) transbronchial needle aspiration (EBUS-TBNA) is a diagnostic method used to evaluate mediastinal lymphadenopathies, including lung cancer and non-malignant diseases, such as tuberculosis and sarcoidosis [1–4]. The diagnostic yield of EBUS-TBNA is reported to be 93–97% [4], which is comparable with that of surgical mediastinoscopy; however, EBUS-TBNA has some advantages over mediastinoscopy. EBUS-TBNA is a minimally invasive procedure, and it is possible to approach hilar/interlobar lymph nodes (LNs) and retrocardiac LNs that cannot be accessed using mediastinoscopy [5].

According to guidelines addressing mediastinal staging in lung cancer, discrete LN(s) on chest computed tomography (CT) with high 18F-fluorodeoxyglucose (FDG) uptake on positron emission tomography (PET) should be visualized for accurate staging [6]. However, these findings do not necessarily correspond with cytopathological analysis, thus resulting in false-positive results [3, 7]. To overcome this barrier, quantitative CT texture analysis (CTTA) was developed to analyze various imaging findings of the LN itself using specialized software, which provides information beyond that obtained by morphological and size assessment performed using the naked eye.

It has been reported that sonographic features can be useful imaging tools for the evaluation of mediastinal and hilar LNs during EBUS [8–12]. Schmid-Bindert et al. [8] suggested six features including the following: short axis length; heterogeneous pattern; round shape; distinct margin; absence of central hilar structure; and high blood flow. In addition, they reported that heterogeneity was the most powerful feature for prediction of malignant LNs. More recently, Fujiwara et al. [9] suggested four items indicating malignant LNs: round shape, distinct margin, heterogeneous echogenicity, and coagulation necrosis sign (CNS). None of these features were present in 96% of patients, who were determined to have no malignancy.

In typical clinical settings, imaging techniques, such as chest CT and/or PET, are performed before EBUS-TBNA. Thus, detailed assessment of LNs using imaging and ultrasound evaluation based on these findings may contribute to diagnostic accuracy. The purpose of the present study, therefore, was to identify features that help distinguish benign and malignant LNs from mediastinal and/or hilar LNs explored using EBUS-TBNA, and to assess the utility of CTTA in this regard.

## Methods

### Patients

**Fig. 1:**
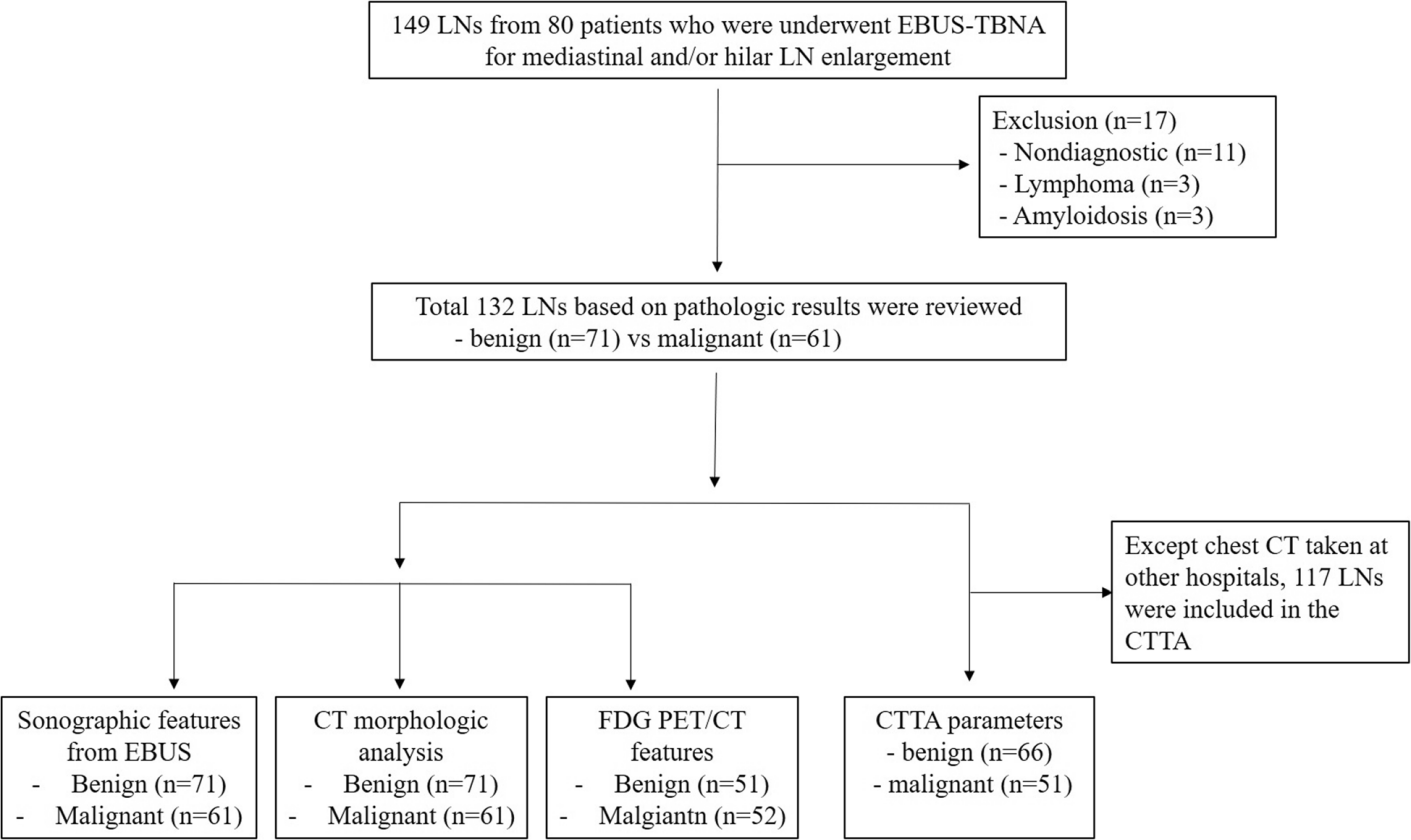
Study flow. CT, computed tomography; CTTA, computed tomography texture analysis; FDG PET/CT, fluorodeoxyglucose-positron emission tomography/CT; EBUS-TBNA, endobronchial ultrasound-guided transbronchial needle aspiration; LN, lymph node

A retrospective review of medical records was performed among patients who underwent EBUS-TBNA for evaluation of mediastinal and/or hilar LN(s), either for staging of lung cancer or lymphadenopathy alone, at the Department of Pulmonology, KyungHee University Hospital, (KHUH, Seoul, Korea) between March 2017 and July 2018. EBUS-TBNA was performed for patients with radiologically identified mediastinal and/or hilar LNs on enhanced chest CT imaging. LN stations were determined according to the International Association for the Study of Lung Cancer (IASLC) guideline [13]. Cases with only primary mass punctures were excluded from this study. LNs classified as benign or malignant according to pathological results were included in the analysis according to chest CT, PET/CT, CTTA, and EBUS features (Fig. 1). The present study was approved by the Ethics Review Board of KHUH (IRB No., KHUH 2018–08-023).

### EBUS-TBNA

EBUS-TBNA was performed on an inpatient basis with patients under conscious sedation (midazolam and fentanyl) using local anesthesia with lidocaine. Midazolam is routinely infused at 0.05 mg/kg, and fentanyl (25 mcg) was administered before commencing EBUS-TBNA. If sedation was insufficient or the patient was irritable or awake during the procedure, an additional 1–2 mg dose of midazolam was administered. A real-time linear probe (BF-UC260FW; Olympus, Tokyo, Japan) was used for EBUS and a 22-gauge needle (NA-201SX-4022; Olympus) for TBNA. After white-light bronchoscopic evaluation, the EBUS scope, equipped with a convex probe, was inserted. LN location was determined based on the standard LN map, and all procedures were performed by a single expert bronchoscopist. The number of punctures and decisions regarding procedure completion were at the discretion of the operator according to the clinical situation.

### EBUS image characteristics of LNs

All EBUS images were assessed by a single attending pulmonologist, who performed all EBUS-TBNA in this study immediately after completion of the procedure in the bronchoscopy room. LN features were categorized based on the EBUS images [8, 9]: short axis diameter (< 1 cm or ≥ 1 cm); shape (round or oval); margin (distinct or indistinct); echogenicity (homogeneous or heterogeneous); central hilar structure (absent or present); and CNS (absent or present) (See Additional file 1 Figure S1).

### CT scanning protocol and CT image analysis

Chest CT images were acquired using 128-detector (Ingenuity Core, Philips Healthcare), 64-detector (Brilliance, Philips Healthcare), or 160-detector (Aquillion Prime, Toshiba Healthcare) scanners. Scan parameters included the following: power, 120 kV; and current, 100–400 effective mA with dose modulation. Reconstruction intervals were 2 mm thick, which is a 2 mm interval without a gap. Enhanced CT images were acquired using a 45-s delay after intravenous administration of 100 mL noninonic contrast medium (hexosure [iohexol], LG, South Korea) and 50 mL of normal saline at a rate of 2.4 mL/s using a power injector. All images were viewed on the mediastinal (window width, 450 Hounsfield unit [HU]; window level, 50 HU) and lung window (window width, 1500 HU; window level, − 700 HU) settings on the axial and coronal images on the picture archiving communicating system (PACS).

An experienced thoracic radiologist retrospectively reviewed all CT images. CT images were analyzed using a slice thickness as thin as possible (CT images with 2-mm slice thickness were acquired in the authors’ hospital [135 LNs from 68 patients]; CT images with 2.5-mm to 5-mm slice thickness were acquired at other hospitals [14 LNs from 12 patients]). For CTTA, CT images acquired at the authors’ hospital were analyzed, except images acquired at other hospital.

Using axial and coronal CT scans, the following morphological features were assessed: size (the maximal short- and long-axis diameters); shape (oval or round); margin (well-defined or ill-defined); presence of internal components, such as calcification, fat, or necrosis; and nodal density (HU). The location of the LN was also described: right, left or subcarina, and ipsilateral or contralateral, according to the primary lung cancer.

### Quantitative CTTA

**Fig. 2:**
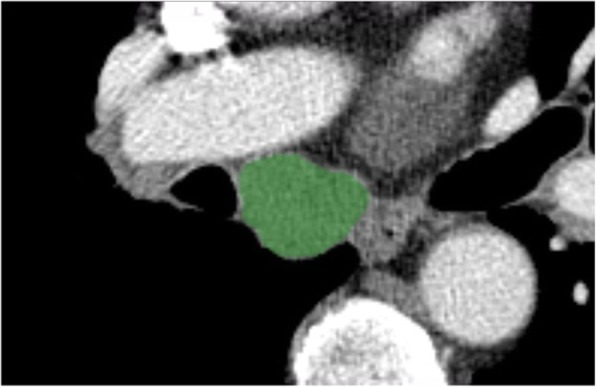
Representative computed tomography image illustrating a region of interest (ROI) demonstrating the enlarged subcarinal lymph node. The green highlighted area represents the ROI

CTTA was performed using commercially available software (AVIEW Research, Coreline Soft, Republic of Korea) by drawing a region of interest (ROI) on a single image with the maximal nodule dimension in the axial plane (Fig. 2). ROIs with calcifications, vessels, and areas related to beam hardening artifact(s) were excluded from the ROIs. After drawing the ROI on an axial image, the following histogram features were obtained from the LNs: mean attenuation; standard deviation (SD); skewness; kurtosis; entropy; and CT attenuation values at the 10th, 25th, 50th, 75th, 90th, and 95th percentiles. Skewness refers to asymmetry of the histogram and calculated as $\mathrm{E}\left[{\left(\frac{X-\mu }{\sigma}\right)}^3\right]$, in which X = attenuation, *σ* = mean of attenuation, and *μ* = standard deviation of attenuation. Kurtosis means the magnitude of the distribution and calculated as $\mathrm{k}=\frac{E{\left(x-\mu \right)}^4}{\sigma^4}-3$, in which x = attenuation, *σ* = mean of attenuation, and *μ* = standard deviation of attenuation. Entropy refers to the irregularity or complexity of the lesion, and calculated as ${\sum}_{i=1}^np(xi)\kern0.24em \log p(xi)$, in which *x*_*i*_ = frequency from histogram of ROI and *p*(*x*_*i*_) = probability on histogram [14, 15]. SD can be affected by size, especially in small lesions; thus, size normalized SD [16] (nSD: SD/N [total number of pixels within the ROI]) was additionally analyzed, which calculated as $\frac{Ln(SD)}{Ln(N)}$.

### ^18^FDG PET/CT acquisition protocol

^18^F-FDG PET/CT was performed using a Gemini TF16 PET scanner (Philips Healthcare, Cleveland, OH), which has an intrinsic resolution of 4.8 mm full-width at half-maximum and simultaneous imaging of 50 contiguous transverse planes with a thickness of 4 mm for a longitudinal field of view of 18 cm. All patients fasted for at least 6 h before PET imaging. Imaging was initiated using a planar scout scan to define the axial range of the study, followed by volumetric CT acquisition. CT parameters were 120 kVp, 50 mAs, and 2-mm slice width and separation. A 1-min emission scan per bed position was performed after the intravenous injection of ^18^F-FDG of 296 MBq. All emission scans were performed in three-dimensional acquisition mode. Attenuation-corrected images were reconstructed into a 144 × 144 matrix by means of an ordered subset expectation maximization algorithm incorporating TOF information (TOF-OSEM). The reconstruction parameters for TOF-OSEM were 3 iterations and 33 subsets.

### FDG PET/CT image analysis

FDG PET/CT is the most widely used non-invasive diagnostic modality for detection of primary tumor(s) and metastasis [17]. Maximum and peak standardized uptake value (SUV) and HU of the LNs were measured using MIRADA software using ROI covering the LNs. SUVs alone and SUVmax ≥2.5 and HU < 70 were used as the criteria to diagnose LNs [18].

### Statistical analysis

Pearson’s chi-squared test for categorical variables, and the Student’s t-test or Fisher’s exact test for continuous variables, were used to compare baseline characteristics, and are expressed as absolute number with percentages, or mean ± standard deviation, unless otherwise indicated. All LNs were categorized according to the final pathology report. To determine which features of various imaging modalities, including chest CT, quantitative CTTA, PET/CT and EBUS, were the most relevant to distinguish benign and malignant LNs, area under the receiver operating curve (AUROC) analysis was performed. A classification and regression tree (CART) analysis—a type of decision-tree methodology for the identification of mutually exclusive subgroups of at-risk individuals who share common characteristics related to particular health-related behavior—was also performed. Sensitivity and specificity were calculated for various radiological features.

All analyses were two-sided, and *P* < 0.05 was considered to be statistically significant. All analyses were performed using STATA version 14.2 (StataCorp, College Station, TX, USA).

## Results

### Patient characteristics

Among 80 patients who underwent EBUS-TBNA for mediastinal and/or hilar LN enlargement, 149 LNs were examined. The mean age of the cohort was 69 years, males were predominant (*n* = 53 [66.3%]), and former smokers accounted for 70%, with a mean history of 40 pack-years. The most common purpose of the EBUS-TBNA procedure was to diagnose or stage lung cancer. A mean of 2 LNs were punctured per patient; subcarinal (7) and right lower paratracheal (4R) LNs were obtained in 31.5 and 27.5% of cases, respectively, and left lower paratracheal (4 L), right upper paratracheal (2R), right hilar (10R) and left hilar (10 L) LNs were followed (Table 1).

**Table 1:** Patient characteristics

Characteristic	Value
Patients, n	80
Age (years)	69 (60–77)
Male sex	53 (66.3)
Smoking status	
Never smoker	24 (30.0)
Ex-smoker	32 (40.0)
Current smoker	24 (30.0)
Smoking pack-year	40 (25–50)
Underlying condition	
Previous malignancy	
NSCLC	7 (8.8)
Extra-thoracic malignancy	11 (13.8)
Lymphoma	3 (3.8)
Interstitial lung disease	2 (2.5)
Previous history of pulmonary tuberculosis	4 (5.0)
COPD	18 (22.5)
Purpose of EBUS-TBNA	
Lung cancer staging and/or diagnosis	65 (81.3)
Metastasis from extra-thoracic malignancy	10 (12.5)
Lymphoma	2 (2.5)
Sarcoidosis	3 (3.7)
Examined lymph node, total	149
Examined lymph node per patients	2 (1–3)
Nodal station^*^	
2R	14 (9.4)
2 L	1 (0.7)
4R	41 (27.5)
4 L	18 (12.1)
7	47 (31.5)
10R	9 (6.0)
10 L	9 (6.0)
11R	5 (3.4)
11 L	2 (1.3)
3P: paraesophageal	2 (1.4): 1 (0.7)
SUV_max_ on FDG PET/CT^†^	5.0 (2.3–6.3)

Data presented as n (%) or median (interquartile range), unless otherwise indicated

^*^2R, right upper paratracheal; 2 L, left upper paratracheal; 4R, right lower paratrachea; 4 L, left lower paratracheal; 7, subcarina; 10R, right hilar; 10 L, left hilar; 11R, right interlobar; 11 L, left interlobar; 3P, retrotracheal

^†^18-Fluorodeoxyglucose positron emission tomography/computed tomography (FDG PET/CT) results were available from 103 lymph nodes in 57 patients

COPD, chronic obstructive pulmonary disease; EBUS-TBNA, endobronchial ultrasound-guided transbronchial needle aspiration; NSCLC, non-small cell lung cancer; SUV_max_, maximal standardized uptake value

The final diagnoses according to cytological and histological results are summarized in Table 2. Lung cancers, including non-small cell lung cancer (NSCLC) and small cell lung cancer, were diagnosed in 35.5% of cases, while reactive hyperplasia was diagnosed in 36.2%. Among 149 LNs, 13 were classified as nondiagnostic due to the presence of a bloody smear only or paucicellularity. After exclusion of cases involving lymphoma, amyloidosis and nondiagnostic specimens, 132 LNs were classified into two groups: malignant (*n* = 61) and benign (*n* = 71).

**Table 2:** Final diagnosis according to cytological and pathological results of lymph nodes (*n* = 149)

Diagnosis	n (%)
Lung cancer	
NSCLC	53 (35.5)
Squamous cell carcinoma	16 (10.7)
Adenocarcinoma	20 (13.4)
SCLC	16 (10.7)
Neuroendocrine carcinoma	1 (0.7)
Metastasis from extrathoracic malignancy	6 (4.0)
Lymphoma	3 (2.0)
Tuberculosis	1 (0.7)
Sarcoidosis	4 (2.7)
Amyloidosis	3 (2.0)
Reactive hyperplasia	54 (36.2)
Nonspecific inflammation	5 (3.4)
Fungal infection	1 (0.7)
Necrosis	4 (2.7)
Leiomyoma	2 (1.3)
Non-diagnostic	13 (8.9)

*NSCLC*, Non-small cell lung cancer; *SCLC*, Small cell lung cancer

### Sonographic findings of EBUS

A short-axis diameter, measured from the ultrasound reference line as > 1 cm, was more frequently found in malignant than in benign LNs (57.8% versus [vs] 88.5%; *P* < 0.001). A round shape and heterogeneous echogenicity were more common in malignant LNs (39.4% vs 59.0 and 11.3% vs 37.7%; *P* < 0.05 for both). CNS was also encountered more frequently in malignant LNs (1.4% vs 9.8%; *P* = 0.031) (Table 3).

**Table 3:** Sonographic features of benign and malignant lymph nodes (LNs)^*^

Characteristic	Benign	Malignant	*P* value
Included lymph nodes	71 (53.8)	61 (46.2)	
Sonographic features			
Size, ≥1 cm	41 (57.8)	54 (88.5)	< 0.0001
Shape: round	28 (39.4)	36 (59.0)	0.025
Margin, distinct	67 (94.4)	60 (98.4)	0.231
Echogenicity:			
Homogeneous	52 (88.7)	38 (62.3)	< 0.0001
Heterogeneous	8 (11.3)	23 (37.7)	
Central hilar structure, present	48 (67.6)	32 (52.5)	0.076
Coagulation necrosis sign, present	1 (1.4)	6 (9.8)	0.031

Data presented as n (%) unless otherwise indicated

*Only cytologically and pathologically confirmed malignant and benign LNs were included, except for lymphoma, amyloidosis, and non-diagnostic specimens

### Two-dimensional and textural features of chest CT

Various chest CT parameters and CT textural features are summarized in Table 4. Seventy-one benign and 61 malignant LNs were included in the CT parameter analysis. LNs located in the same direction as the primary tumor were more prevalent in malignant LNs and, those in the opposite direction were more prevalent in benign LNs; however, the difference was not statistically significant. It was not possible to assess the position of 13 benign and 7 malignant LNs due to sarcoidosis and tuberculosis lymphadenopathy involved in the LN alone. Short-axis diameter was longer in malignant compared with benign LNs (15.7 mm vs 11.2 mm, respectively; *P* < 0.001). Shape, well-demarcated margin, and HU between benign and malignant LN were not significantly different.

**Table 4:** Morphological computed tomography (CT), CT texture analysis (CTTA), and 18-fluorodeoxyglucose positron emission tomography/CT (FDG PET/CT) parameters for malignant and benign lymph nodes

	Benign	Malignant	*P* value
Morphological CT parameters, including lymph nodes, n	71	61	
Location of primary tumor and lymph node			
Ipsilateral	25 (50.7)	40 (65.6)	0.384
Contralateral	19 (26.8)	12 (19.7)	
Subcarinal	3 (4.2)	2 (3.3)	
Short axis diameter, mm	11.2 ± 4.2	15.7 ± 6.3	< 0.0001
Long axis diameter, mm	16.2 ± 5.2	22.2 ± 7.6	< 0.0001
Shape, round	7 (9.9)	10 (16.4)	0.264
Margin, well-defined	64 (90.1)	52 (85.3)	0.390
Noticeability (> 60 HU)	52 (73.2)	38 (62.3)	0.178
CT texture parameters, including lymph nodes	66	51	
Area (mm^2^)	160.3 ± 111.1	338.8 ± 263.5	< 0.0001
Circularity	0.84 ± 0.07	0.83 ± 0.07	0.358
Compactness	0.97 ± 0.01	0.98 ± 0.01	0.003
Roundness	0.76 ± 0.08	0.77 ± 0.07	0.544
Entropy	6.6 ± 0.5	6.8 ± 0.5	0.142
Mean HU	69.6 ± 23.5	68.3 ± 23.0	0.774
Standard deviation (SD)	43.2 ± 19.6	38.0 ± 13.6	0.108
Normalized SD^*^	0.19 ± 0.13	0.10 ± 0.08	< 0.0001
Skewness	−0.00 ± 0.02	0.00 ± 0.02	0.562
Kurtosis	3.1 ± 0.7	3.2 ± 0.9	0.914
10th percentile HU	11.8 ± 31.9	19.3 ± 26.3	0.174
25th percentile HU	41.2 ± 24.7	42.2 ± 23.2	0.816
50th percentile HU	71.7 ± 23.0	68.1 ± 23.2	0.397
75th percentile HU	101.0 ± 28.0	94.4 ± 26.4	0.194
90th percentile HU	125.0 ± 36.3	116.4 ± 32.1	0.185
FDG PET/CT parameter, Included lymph nodes	51	52	
SUV_max_	3.4 ± 2.4	6.3 ± 3.8	< 0.0001
SUV_Peak_	2.7 ± 1.6	5.2 ± 3.1	< 0.0001
Maximal HU^†^	119.1 ± 283.5	80.4 ± 147.3	0.385
SUV_max_ ≥ 2.5 and HU < 70	15 (29.4)	40 (76.9)	< 0.0001

Data presented as n (%) or mean ± standard deviation, unless otherwise indicated

*Normalized standard deviation (SD) is calculated as In(SD)/In(n)[=In(SD)/In(total number of pixels within the region of interest)]

*HU*, Hounsfield unit; *SUV*, standard uptake value

^†^ *HU* values obtained from non-enhanced chest *CT* performed with FDG PET/CT

A total of 117 LNs were included in CTTA, except CT, which included only non-contrast images acquired at external hospitals with low quality that did not match the chest CT at KHUH. CTTA was evaluated in 66 benign and 51 malignant LNs. Compactness and normalized SD were significantly different between the benign and malignant LNs (*P* < 0.001 for both). There were no significant differences noted in the remaining CTTA parameters including mean HU, entropy, skewness, and kurtosis.

### FDG PET/CT results

Maximum and peak SUV were significantly higher in malignant than in benign LNs (6.3 vs 3.4; and 5.2 vs 2.7, respectively; *P* < 0.001 for both). Although maximal HU was higher in benign LNs, the difference was not statistically significant (Table 4). Peak SUV value ≥4 and maximal SUV value ≥5 were good determinants of malignant LNs in the CART analysis (relative hazard risk, 1.70 and 1.69, respectively). The combined PET/CT parameters of SUV_max_ ≥ 2.5 and HU < 70 was significantly higher in malignant than benign LNs (76.9% vs 29.4%, respectively).

### Diagnostic yield of the imaging modalities

The diagnostic accuracy and sensitivity of various features extracted from chest CT, EBUS, CTTA and PET/CT are shown in Table 5. ROC analyses revealed that normalized SD demonstrated the highest accuracy for differentiating benign and malignant LNs when benign was encoded as 1 and malignant LN as 0, unlike other parameters (AUC, 0.742). High maximal SUV (≥5) and high peak SUV (≥4) on PET/CT demonstrated similar ability to distinguish benign and malignant LNs (AUC, 0.739 for both), followed by compactness on CTTA (AUC, 0.666), short axis diameter > 10 mm on chest CT and EBUS (AUC, 0.659 and 0.654, respectively), and heterogenic echogenicity (AUC, 0.632).

**Table 5:** Receiver operating characteristic curve analysis of chest computed tomography (CT), textural features, 18-fluorodeoxyglucose positron emission tomography/CT (FDG PET/CT) and sonographic findings

Variable	AUC	SE (AUC)	95% CI	Se (%)	Sp (%)
Short-axis diameter, ≥ 10 mm	0.659	0.038	0.585–0.732	93	58.7
Compactness on CTTA	0.666	0.051	0.566–0.766	10.6	100
Normalized SD on CTTA^†^	0.742	0.046	0.652–0.833	8.2	100
FDG PET/CT					
SUV_max_ ≥ 2.5	0.639	0.042	0.556–0.721	94.4	55.8
SUV_max_ ≥ 5	0.739	0.042	0.657–0.821	74.7	94.3
SUV_Peak_ ≥ 4	0.739	0.041	0.658–0.820	73	95.6
SUV_max_ ≥ 2.5 and HU < 70	0.738	0.044	0.652–0.823	87.5	82.5
Size on EBUS, ≥10 mm	0.654	0.036	0.583–0.724	95.3	54.6
Round shape	0.598	0.043	0.513–0.682	71.4	72
Heterogeneous echogenicity	0.632	0.037	0.561–0.704	51	95
Coagulation necrosis sign	0.542	0.021	0.502–0.582	20.2	100

*AUC*, Area under the receiver operating characteristic curve; *CI*, Confidence interval; *EBUS*, Endobronchial ultrasound; *SD*, Standard deviation; *Se*, Sensitivity; *Sp*, Specificity; *SUVmax*, Standard maximal uptake value

^†^Analyses was performed using 1 = benign and 0 = malignant

However, compactness and normalized SD on CTTA had very low sensitivity, despite high specificity. LN size demonstrated low specificity and, echogenic characteristics, such as echogenicity and the presence of CNS, had relatively low sensitivity. Only PET/CT criteria combining SUV_max_ ≥ 2.5 and HU < 70 had high sensitivity (87.5%) and specificity (82.5%) for malignancy.

### Diagnostic yield of combining features of chest CT and PET/CT

Diagnostic accuracy and sensitivity analyses were also performed by combining three two-dimensional chest CT and CTTA features (short-axis diameter ≥ 10 mm, compactness and normalized SD) and that of PET/CT as well (SUV_max_ ≥ 2.5 and HU < 70, SUV_max_ ≥ 5 and SUV_peak_ ≥ 4) (Additional file 2 Table S1). Several combinations of each item of chest CT and CTTA did not achieve better diagnostic accuracy. The combination of CT features and SUV, either maximal or peak value, also did not yield better accuracy, and only size on two-dimensional chest CT and the combination of SUVmax ≥2.5 and HU < 70 demonstrated slightly higher diagnostic accuracy.

## Discussion

We analyzed imaging features of various modalities, including chest CT, PET/CT, CTTA and EBUS, used to distinguish malignant and benign LNs, which were pathologically identified using EBUS-TBNA, and evaluated their diagnostic accuracy. In addition to established findings in malignant LNs, such as size, shape, echogenicity, CNS in EBUS, size on chest CT, high SUV in PET/CT, compactness, and normalized SD on CTTA were features that distinguished malignant and benign LNs. Although normalized SD on CTTA demonstrated high diagnostic accuracy, the sensitivity was low, which suggests it should not be used as a sole diagnostic feature.

Mediastinal LN staging plays a pivotal role in staging and treatment planning in lung cancer. Traditionally, chest CT and PET/CT have been used to predict malignancy. However, a meta-analysis comparing the accuracy of PET/CT and chest CT in detecting mediastinal LN metastases in patients with NSCLC reported that PET/CT was more accurate than chest CT in distinguishing metastatic mediastinal LNs [7]. However, there is the possibility of false-positive results when evaluating only FDG uptake on PET/CT, and it has been reported that it is more likely to be benign LN, with higher attenuation, than surrounding vessels or calcified LNs [19, 20]. Lee et al. [21] reported that integrated SUVmax and LN density criteria demonstrated higher accuracy for characterizing metastatic NSCLC. We compared the criteria based on SUVmax ≥2.5, widely known as a positive standard [22, 23], and ≥ 5 with high discriminatory power in our study. Criteria combining SUVmax ≥2.5 and HU < 70 [18] in our study revealed that combined PET/CT criteria demonstrated higher diagnostic accuracy than SUVmax alone, and similar accuracy with higher SUVmax (≥5). Nevertheless, a Cochrane systematic review, including 45 studies that assessed the diagnostic accuracy of PET/CT for diagnosing N2 disease in patients with resectable NSCLC, reported sensitivity and specificity estimates for the SUVmax ≥2.5 PET/CT positivity criteria were 81.3% (95% CI 70.2–88.9) and 79.4% (95% CI 70–86.5), respectively; however, they noted high between-study heterogeneity and lack of precision [24]. This result reflects the insufficiency of PET/CT alone to guide management.

In chest CT, beam hardening artifacts may occur from contrast media in the vessel(s) near the LNs, which tend to cause artifacts in CT morphology, diameter assessment, and CTTA. This may lead to poor measures, and this limitation of CT imaging can be overcome by LN evaluation using real-time EBUS. There are some established sonographic features suggestive of metastatic LN, including short axis length, round shape, distinct margin, heterogenic echogenicity, and absence of CNS [8–10, 25]. However, previous studies have reported that sonographic features, even after some feature integration, favor metastatic LNs, although with low sensitivity and specificity [25].

Occasionally, small-size LNs, which are not visible on chest CT or PET/CT, can be observed during EBUS. A dilemma, however, can arise between clinical significance and the risk associated with the procedure as to whether to puncture these small LNs. There is no guidance for these LNs, and the issues of procedural time and specialized personnel are also involved. In this context, there has been an urgent need for an imaging modality that can accurately predict malignant potential. When lung cancer is evaluated in clinical practice, imaging tests, including chest CT and PET/CT, are usually performed ahead of EBUS-TBNA. Although LN(s) observed using these image modalities and EBUS are correlated, there is only limited agreement [26]. CTTA has been developed to complement the limitations of existing image modalities, and to enable more accurate malignancy risk prediction.

Digumarthy et al. [14] compared 58 benign and 120 malignant LNs using CTTA; normalized SD had moderate accuracy for differentiating benign LNs from those that were malignant (AUC 0.63 [95% CI 0.55–0.72]; *P* = 0.003). Bayanati et al. [27], evaluated 72 LNs from 43 patients, and reported that CTTA had the potential to accurately differentiate malignant and benign mediastinal LNs in lung cancer. They reported that combining six features of CTTA enabled identification of malignant mediastinal nodes with 81% sensitivity and 80% specificity (AUC, 0.87; *P* < 0.0001). Additionally, Andersen et al. [28], reported that mean image intensity values obtained from CTTA, for identification of LNs likely to be malignant, classified 82.6% of the cases correctly (53% sensitivity and 97% specificity) in 46 LNs from 29 patients. The former two studies used TexRAD research software (TexRAD Ltd., Cambridge, United Kingdom), while the latter used MaZda version 4.6 (Institute of Electronics, Technical University of Lodz, Poland). In this study, we used AVIEW (Corelinee Soft, Seoul, Korea) and compared the diagnostic accuracy with other imaging modalities that are mandatory for staging of lung cancer. The degree of agreement among CTTA software was not studied, nor was itemized standardization achieved.

The important implication of our results is that 2 CTTA parameters—compactness and normalized SD—differed between benign and malignant LNs. Compactness, which is defined as the ratio of the square of surface area to the total volume of the lesion, is a morphological feature and has been reported to have a higher value in malignant cancers [29, 30], and normalized SD, which is linked to entropy and known to be useful for smaller ROIs, has recently been reported to discriminate malignant from benign LNs [14]. Although normalized SD has a moderate ability to discriminate benign from malignant LNs (AUC, 0.742), it has low clinical utility (sensitivity 8.2%, specificity 100%). Moreover, even combining two features in CTTA with PET/CT features, which are known predictors for distinguishing malignant from benign LN, could not achieve better diagnostic accuracy.

In the present study, we sought to identify a parameter to distinguish malignant and benign mediastinal LNs in patients with suspected lung cancer based on pathologically confirmed results by comparing the diagnostic accuracy of various imaging modalities and CTTA. However, there are several important limitations to consider. First, we included a relatively small number of patients and LNs, for whom pathologically proven lymphoma and amyloidosis were excluded from the analysis due to other malignant features different from lung cancer. However, it is difficult to rule out the possibility that this exclusion caused selection bias. Second, it is possible that selection bias was introduced because we analyzed CT images with a thin slice thickness and excluded images acquired from outside hospitals for CTTA. Additionally, there is also the possibility of observer bias, because the segmentation of LNs on CT was performed by a single radiologist. Third, this study was performed in a single institution, and all included EBUS-TBNA was performed by one bronchoscopist with significant experience. Even a small LN with a short axis diameter of 4 mm was punctured during EBUS-TBNA, which may be difficult to generalize to other settings. Finally, a standardized CTTA is not yet available for clinical practice and is only being explored for research purposes. During the past few years, many studies have addressed the potential of radiomics. By reviewing these investigations and analyzing data from our study, we agree that the implementation of radiomics in clinical settings remains limited and believe it will be necessary to find other parameters to address its shortcomings. Moreover, we believe that variable background texture (i.e., lung, mediastinum, breast) and other factors, such as beam hardening artifact(s), field of view, lesion size and interobserver variability, among others, may affect the results, and that further studies focusing on compensation tools are warranted.

## Conclusion

In this study, we found that some CTTA parameters may help to distinguish between malignant and benign LNs; however, CTTA-guided clinical decisions remain difficult. Therefore, CTTA can only be used as a supplementary aid due to its relatively low diagnostic value. It is necessary to evaluate the clinical significance of LNs by integrating various features on other image modalities because CTTA itself is a novel technique, and further studies are needed.

## Additional file

Additional file 1: **Figure S1.** Endobronchial ultrasound (EBUS) findings. Round shape (a), oval shape (b), central hilar structure (CHS) (c), homogeneous (d) and heterogeneous (e) echogenicity, and coagulation necrosis sign (CNS) (f). (a) Round shape was defined when the ratio of the short- to long-axis diameter of lymph nodes was ≥1.5 and (b) oval shape was defined as when the ratio was < 1.5. (c) CHS was defined as a linear, flat, hyperechoic area in the center of the lymph node. (d) Distinct margin was defined when the majority of the margin (> 50%) was clearly visualized with a high echoic border and, if the margin was unclear, the lymph node was assessed with an indistinct margin. (e) Heterogeneous echogenicity was defined as multiple low echoic spots within the lymph node. (f) The CNS is a hypoechoic area within the lymph node without blood flow. (TIFF 850 kb)

Additional file 2: **Table S1.** Receiver operating characteristic curve analysis combining features of chest computed tomography (CT), textural features, and 18-fluorodeoxyglucose positron emission tomography/CT (FDG PET/CT) (DOCX 18 kb)

## Abbreviations

AUROC: Area under the receiver operating curve (AUROC)
CART: Classification and regression tree
CNS: Coagulation necrosis sign
CT: Computed tomography
CTTA: Computed tomography texture analysis
EBUS-TBNA: Endobronchial ultrasound-guided transbronchial needle aspiration
FDG: Fluorodeoxyglucose
LN: Lymph node
NSCLC: Non-small-cell lung cancer
PET: Positron emission tomography
