# Supplementary figures and images for: Quantitative computed tomography texture analysis: can it improve diagnostic accuracy to differentiate malignant lymph nodes?
Source: Cancer Imaging. 2019 May 22;19:25. doi: 10.1186/s40644-019-0214-8 (PMC6530003; doi:10.1186/s40644-019-0214-8)

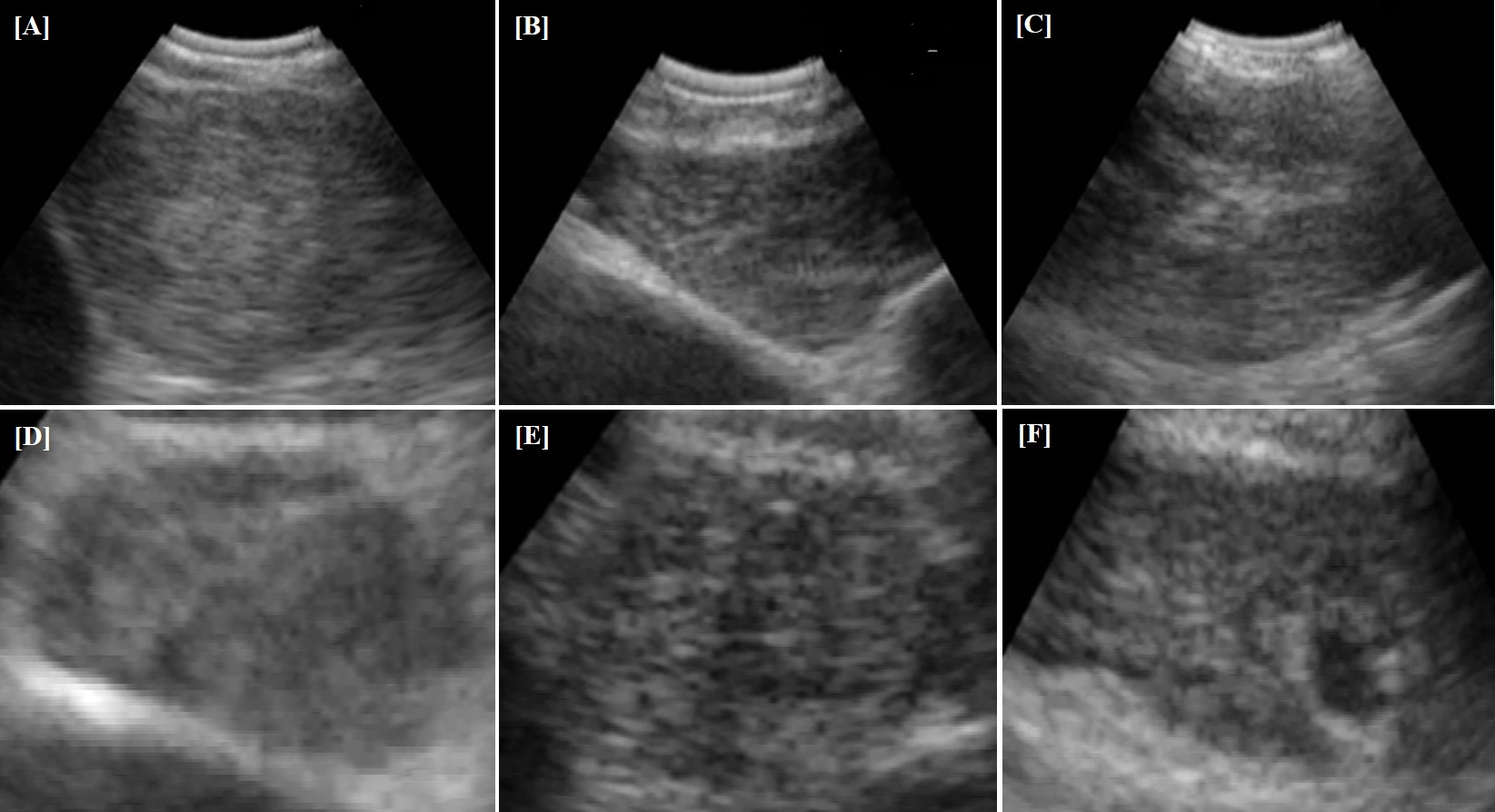

Supplement: Supplementary file 1 — Figure S1. Endobronchial ultrasound (EBUS) findings. Round shape (a), oval shape (b), central hilar structure (CHS) (c), homogeneous (d) and heterogeneous (e) echogenicity, and coagulation necrosis sign (CNS) (f). (a) Round shape was defined when the ratio of the short- to long-axis diameter of lymph nodes was ≥1.5 and (b) oval shape was defined as when the ratio was < 1.5. (c) CHS was defined as a linear, flat, hyperechoic area in the center of the lymph node. (d) Distinct margin was defined when the majority of the margin (> 50%) was clearly visualized with a high echoic border and, if the margin was unclear, the lymph node was assessed with an indistinct margin. (e) Heterogeneous echogenicity was defined as multiple low echoic spots within the lymph node. (f) The CNS is a hypoechoic area within the lymph node without blood flow. (TIFF 850 kb) [file 40644_2019_214_MOESM1_ESM.tiff]
