# Supplementary material for: Quantitative computed tomography texture analysis: can it improve diagnostic accuracy to differentiate malignant lymph nodes?
Source: Cancer Imaging. 2019 May 22;19:25. doi: 10.1186/s40644-019-0214-8 (PMC6530003; doi:10.1186/s40644-019-0214-8)
Supplement: Supplementary file 2 — Table S1. Receiver operating characteristic curve analysis combining features of chest computed tomography (CT), textural features, and 18-fluorodeoxyglucose positron emission tomography/CT (FDG PET/CT) (DOCX 18 kb) [file 40644_2019_214_MOESM2_ESM.docx]

| Variable | AUC | SE (AUC) | 95% CI | Se (%) | Sp (%) |
| --- | --- | --- | --- | --- | --- |
| Combination of chest CT and CTTA features | | | | | |
| (A) & (B) | 0.638 | 0.041 | 0.558-0.718 | 85.5 | 64.1 |
| (A) & (C) | 0.647 | 0.040 | 0.569-0.726 | 62.3 | 88.8 |
| (A + B + C) | 0.623 | 0.040 | 0.544-0.701 | 57.6 | 88.8 |
| Combination of chest CT, CTTA and PET/CT features | | | | | |
| (A) & (D) | 0.764 | 0.039 | 0.687-0.841 | 75.2 | 96.9 |
| (B) & (D) | 0.675 | 0.036 | 0.605-0.746 | 54.1 | 99.0 |
| (C) & (D) | 0.648 | 0.035 | 0.579-0.717 | 48.6 | 99.0 |
| (A) & (E) | 0.717 | 0.039 | 0.640-0.794 | 64.9 | 97.9 |
| (B) & (E) | 0.628 | 0.031 | 0.567-0.690 | 41.0 | 100.0 |
| (C) & (E) | 0.619 | 0.031 | 0.559-0.679 | 39.0 | 100.0 |
| (A) & (F) | 0.717 | 0.039 | 0.640-0.794 | 64.9 | 97.9 |
| (B) & (F) | 0.628 | 0.031 | 0.567-0.690 | 41.0 | 100.0 |
| (C) & (F) | 0.619 | 0.031 | 0.559-0.679 | 39.0 | 100.0 |

Short-axis diameter ≥ 10 mm (A), compactness on CTTA ≥ 0.97 (B), normalized SD<0.09 (C), SUV_max_≥2.5 and HU<70 (D), SUV_max_≥5 (E), and SUV_peak_≥4 (F)

AUC, area under the receiver operating characteristic curve; SE, standard error; Se, sensitivity; SP, specificity
